# Individual and Combined Effect of Bisphenol A and Bisphenol AF on Prostate Cell Proliferation through NF-κB Signaling Pathway

**DOI:** 10.3390/ijms232012283

**Published:** 2022-10-14

**Authors:** Kaiyue Wang, Dongyan Huang, Ping Zhou, Xin Su, Rongfu Yang, Congcong Shao, Aicui Ma, Jianhui Wu

**Affiliations:** 1 NHC Key Lab of Reproduction Regulation (Shanghai Institute for Biomedical and Pharmaceutical Technologies), Pharmacy School of Fudan University, Shanghai 200032, China; 2 Department of Pharmacology & Toxicology, Shanghai Institute for Biomedical and Pharmaceutical Technologies, Shanghai 200032, China

**Keywords:** bisphenols, prostatic hyperplasia, co-exposure, nuclear transcription factor-κB, cyclooxygenase-2

## Abstract

The ubiquitous environmental endocrine disruptor bisphenol A (BPA) can induce prostatic dysfunction. However, to date, studies have focused little on the perturbations of prostate health initiated by the BPA derivative bisphenol AF (BPAF) and co-exposure to bisphenol compounds. An in vivo study orally administrated male rats with BPA (10, 90 μg/kg), BPAF (10, 90 μg/kg) and the inhibitor of nuclear transcription factor-κB (NF-κB), pyrrolidinedithiocarbamate (PDTC, 100 mg/kg). Based on the anatomical analysis, pathological observations and PCNA over-expression, we considered that low-dose BPA and BPAF facilitated ventral prostatic hyperplasia in rats. The results of IHC and ELISA mirrored the regulation of NF-κB p65, COX-2, TNF-α and EGFR in BPA- and BPAF-induced prostatic toxicity. An in vitro study found that the additive effect of combined exposure to BPA (10 nM) and BPAF (10 nM) could cause an elevation in the proliferation of and a reduction in the apoptosis level of human prostate stromal cells (WPMY−1) and fibroblasts (HPrF). Meanwhile, the underlying biomarkers of the NF-κB signaling pathway also involved the abnormal proliferative progression of prostate cells. The findings recapitulated the induction of BPAF exposure and co-treatment with BPA and BPAF on prostatic hyperplasia and emphasized the modulation of the NF-κB signaling pathway.

## 1. Introduction

Bisphenol A (BPA), an exogenous estrogen with endocrine disrupting effects, is widely available for consumer goods, medical devices and electronic devices. Residual BPA has also been detected in surface water, sediment, indoor dust and other environmental media [1,2], as well as human plasma, urine and breast milk [3,4]. BPA could mimic the function of endogenous estrogen and recognize and bind estrogen response elements (EREs), thereby affecting the expression of downstream cytokines and, ultimately, interfering with the hormone balance and hormone-dependent growth and development of the prostate. Epidemiologically, frequent oral BPA has been reported to potentially provoke the chronic inflammation of prostate tissue, which is significantly associated with increased benign prostatic hyperplasia (BPH) risk [5]. In in vivo studies, the male CD-1 mice perinatally exposed to 20 μg/kg/day BPA were more prone to prostate regeneration, abnormal renal function and a swollen bladder [6]. BPA exposure is also predominantly responsible for the vascular congestion and hyperplasia of prostatic epithelium in rats [7] and the upregulated proliferative state of prostate and estrogen receptor-α (ERα) activation in prostatic stromal cells in gerbils [8]. Compelling evidence from our previous research has revealed that BPA (2, 6, 18 μg/kg/day), even at environment-relevant concentrations, could trigger the thickened prostate epithelium, hormonal disturbances and malignant transformation of prostatic hyperplasia in beagle dogs [9]. However, the low-dose BPA-induced prostatic toxicology and regulatory mechanism still remain insufficiently understood and require further consideration and exploration.

As the toxicological effects of BPA have been progressively explored, it has become increasingly recognized that long-term exposure to BPA may have some deleterious effects on the nervous and reproductive systems [10,11,12,13]. For this reason, the use of BPA has been severely restricted or prohibited by a wide range of laws and regulations, thus ushering in a new era in the development and application of analogues of BPA with similar molecular structures and estrogen-like effects. Compared with other BPA analogues, bisphenol AF (BPAF) has been shown to have the strongest affinity for estrogen receptors [14] and the highest overall environmental persistence [15]. Bioaccumulation represented the increment of xenobiotic concentrations in the tissues. Currently, only a small amount of findings on the reproductive toxicity in BPAF-affected males considered that BPAF has the potential to contribute to the retardation of gonadal cells [16], sperm deterioration in parents, and delayed hatching of offspring [17], as well as pituitary–gonad dysfunction [18]. It has been speculated that BPAF, serving as a fluoride of BPA, may pave the way to drive susceptibility to prostatic diseases, which has been predicted in a bioinformatics analysis work [19], while the long-term accumulative deleterious effects on the prostate have been poorly confirmed. Consequently, it is crucial to probe the toxic effects and mechanisms of the prostate that are induced by BPA and its analogue BPAF at low doses.

Mechanistically, stem-progenitor cells in the human prostate gland have been accepted as a direct target for the estrogenic effects of BPA [20]. Specifically, upon inappropriate exposure to BPA, the homeostasis, self-renewal capability and remodeling process of prostate stem cells were perturbed, increasing the stem cell numbers and incurring prostatic disorders [21]. In parallel, BPA and BPAF could be considered both as the activators of ERα and the antagonists of ERβ, disrupting prostate health [22]. Our previous studies demonstrated that the prostaglandin synthase, cyclooxygenase-2 (COX-2), could mediate the progress of prostatic hypertrophy in rats treated with BPA (10, 30, 90 μg/kg/day) [23], accompanying the overexpression of the epidermal growth factor receptor (EGFR) [24]. Moreover, COX-2 and EGFR are traditionally associated with nuclear transcription factor-κB (NF-κB) in inflammatory disease, as the downstream effector [25] and upstream trigger [26], respectively. NF-κB consists of two subunits of the NF-κB family, including Rel A (p65), Rel B (p66), NF-κB1 (p50), NF-κB2 (p52) and c-Rel, which is one of the pivotal modulators involving inflammation and immune response, controlling the transcription and expression of the target genes. Additionally, it is reported that the NF-κB signaling pathway was activated in the human prostatic epithelial and stromal cells of BPH patients, thus serving in the maintenance of cell viability and production of drug resistance [27].

In this research, we comprehensively carry out the experimental validation with 90 male rats to explore the individual induction of BPA and BPAF for prostatic hyperplasia, and the expression of the significant indexes of the NF-κB signaling pathway containing NF-κB p65, COX-2, tumor necrosis factor-α (TNF-α) and EGFR. Furthermore, the variable exogenous endocrine disruptors in the environment were simultaneously exposed, and the joint exposure may lead to different degrees of biological dysfunction. Therefore, the combined effect of BPA and BPAF on human prostate stromal cells and fibroblasts and the regulation of the NF-κB signaling pathway in this effect were verified.

## 2. Results

### 2.1. Anatomical Analysis of Prostates in the Rats Treated with BPA, BPAF and PDTC

**Figure 1:**
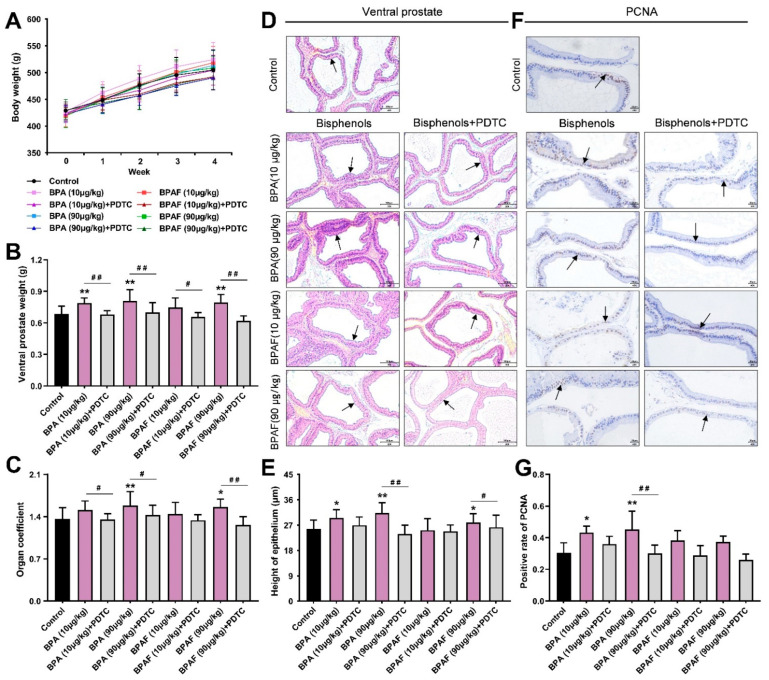
Weight gain and pathological changes of prostates in rats. Effect of exposure to bisphenol A (BPA), bisphenol AF (BPAF) and the inhibitor of nuclear transcription factor-κB (NF-κB), pyrrolidinedithiocarbamate (PDTC), for 4 weeks on body weight (**A**); ventral prostate weight (**B**); and organ coefficient of ventral prostates (**C**), *n* = 10. (**D**) The pathological changes of the tissues (200×, scale bar = 100 μm) and the prostate epithelium are pointed to with arrows. (**E**) The height of prostate epithelium of ventral prostates, *n* = 6. (**F**) The immunohistochemical images of proliferating cell nuclear antigen (PCNA) (400×, scale bar = 20 μm). Arrows indicate the positive expression. (**G**) Immunohistochemistry (IHC) quantification of PCNA was presented as the ratio of the positively expressed nucleus to the total nucleus, *n* = 8. Results were performed as means ± SD, analyzed using ANOVA followed by LSD post hoc test. Comparison of the individual administration group and the control: * *p* < 0.05, ** *p* < 0.01; comparison of the combined administration group and the individual administration group: # *p* < 0.05, ## *p* < 0.01. Organ coefficient = 1000× ventral prostate weight/terminal body weight.

During the 4-week oral administration of BPA, BPAF and the NF-κB inhibitor pyrrolidinedithiocarbamate (PDTC), body weight increased almost linearly, with no significant difference among groups (Figure 1A). We inferred that BPA, BPAF and PDTC may not have significant effects on the normal weight gain in the animals. Anatomical data considered that both the weight of the ventral lobe of the prostate (VP) and the organ coefficient (1000 times the ratio of VP weight to terminal body weight) showed a mild elevation in the BPA (90 μg/kg) group and the BPAF (90 μg/kg) group (Figure 1B,C), reflecting BPA- and BPAF-induced prostate weight gain, respectively. However, concomitant treatment of BPA with PDTC and concurrent exposure to BPAF and PDTC could significantly decrease the weight and organ coefficient of the VP.

### 2.2. BPA- and BPAF-Facilitated Pathological Hyperplasia of the Prostate in Rats

The hematoxylin and eosin staining (H&E) results and the measurement of epithelial height cooperatively mirrored that BPA and BPAF caused the abnormal thickening of the prostate epithelium, the dense distribution of glands and the deformation of acini, while the morphological alternations of the VP induced by BPA and BPAF were alleviated after simultaneous administration of an inhibitor (Figure 1D,E). Serving as the dependable index for the evaluation of proliferation state, the nucleus with positive expression of PCNA could be stained brown in the prostate tissues of each group (Figure 1F). The elevated rate of positive expression of PCNA provided implications that the low-dose BPA could perform the hyperplastic effect in the VP (Figure 1G). Additionally, the BPA or BPAF combined with PDTC lowered the level of PCNA expression in the VP.

### 2.3. Localization and Qualification of the Critical Modulators of NF-κB Signaling Pathway

**Figure 2:**
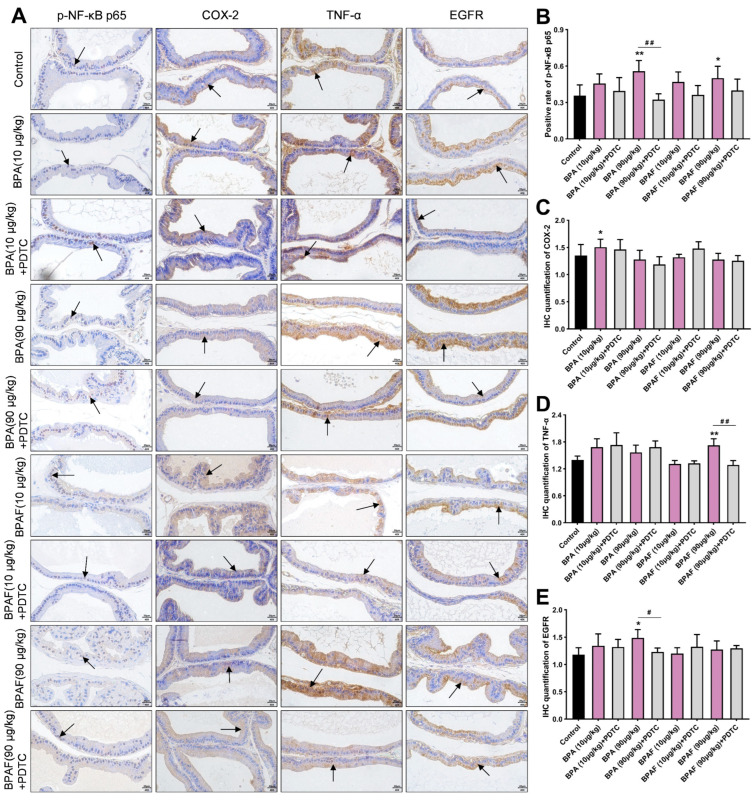
Localization observation of phosphorylated NF-κB p65 (p-NF-κB p65), cyclooxygenase-2 (COX-2), tumor necrosis factor-α (TNF-α) and epidermal growth factor receptor (EGFR) expression in VP. (**A**) Immunohistochemical images of p-NF-κB p65, COX-2, TNF-α and EGFR of the prostate tissues in the rats treated using BPA, BPAF and PDTC (400×, scale bar = 20 μm). Arrows indicate the positive expression. Effect of exposure to BPA, BPAF and PDTC on the rate of positive expression of p-NF-κB p65 (**B**); the semiquantitative expression levels of COX-2 (**C**), TNF-α (**D**) and EGFR (**E**) in ventral prostate (VP). Results were performed as means ± SD, analyzed using ANOVA followed by LSD post hoc test, *n* = 8. Comparison of the individual administration group and the control: * *p* < 0.05, ** *p* < 0.01; comparison of the combined administration group and the individual administration group: # *p* < 0.05, ## *p* < 0.01.

The positive expression of the phosphorylated NF-κB p65 (p-NF-κB p65) and COX-2 were detected in the VP using immunohistochemistry (IHC) (Figure 2A). The unactivated NF-κB was suppressed by its inhibitor (IκB) in cytoplasm. Nevertheless, after ubiquitination degradation of IκB, NF-κB could be phosphorylated and immediately transferred into the nucleus to bind to the specific sites of the target genes [28]. COX-2, TNF-α and EGFR were the hub regulators of the NF-κB signaling pathway and were predominantly expressed in the cytoplasm of the epithelial tissue of the prostate (Figure 2A).

The semiquantitative analysis manifested that BPA and BPAF enhanced the level of positive expression of p-NF-κB p65 and COX-2 in the VP (Figure 2B,C), which revealed that p-NF-κB p65 and COX-2 might be implicated in the prostate enlargement. The significant elevation of TNF-α expression in the VP of the BPAF (90 μg/kg) group and the greater expression of EGFR in the VP of the BPA (90 μg/kg) group cooperatively indicated that TNF-α and EGFR could be inextricably linked with low-dose BPA and BPAF (Figure 2D,E). Additionally, the inhibitor PDTC downmodulated the positive expressions of p-NF-κB p65, COX-2, TNF-α and EGFR in the VP, and the inhibitory effect on p-NF-κB p65 expression was more conspicuous.

### 2.4. BPA and BPAF Stimulated Expressions of NF-κB p65, COX-2, TNF-α and EGFR

**Figure 3:**
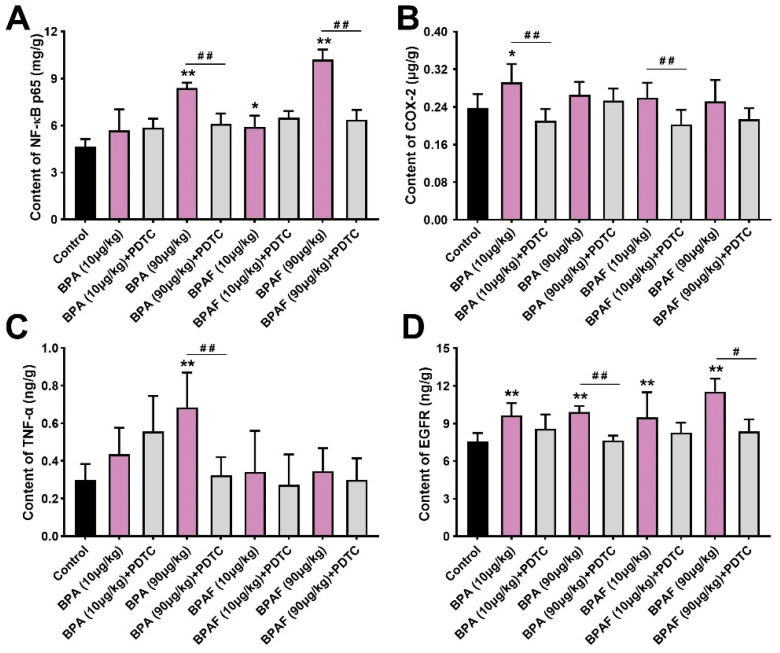
BPA, BPAF and PDTC induced the quantitative changes of p-NF-κB p65, COX-2, TNF-α and EGFR in VP. Content of NF-κB p65 (**A**), COX-2 (**B**), TNF-α (**C**) and EGFR (**D**) was obtained using enzyme-linked immunosorbent assay (ELISA). Results are performed as means ± SD, analyzed using ANOVA followed by LSD post hoc test, *n* = 4. Comparison of the individual administration group and the control: * *p* < 0.05, ** *p* < 0.01; comparison of the combined administration group and the individual administration group: # *p* < 0.05, ## *p* < 0.01.

Similar conclusions were drawn by the tissue-based enzyme-linked immunosorbent assay (ELISA) results (Figure 3A–D). Among the groups, 90 μg/kg BPA had the most significant perturbations on the expressions of NF-κB p65 (Figure 3A). Compared with the control, the content of COX-2 in the VP of the BPA (10 μg/kg) group and the BPAF (10 μg/kg) group demonstrated the ascending trends (Figure 3B). The content of TNF-α was dramatically improved in the VP of the BPA (90 μg/kg) group and was decreased by continuous co-exposure to BPA (90 μg/kg) and PDTC (Figure 3C). Meanwhile, EGFR content ascended in the VP of the low-dose BPA- and BPAF-treated groups, while the PDTC exposure showed a downregulated effect on EGFR levels (Figure 3D). The differences between the semiquantitative analysis using IHC and the quantitative detection using ELISA on the expression of TNF-α and EGFR was, potentially, attributed to methodological differences. Nonetheless, all the above results indicated the BPA- and BPA-associated driver function of TNF-α and EGFR.

### 2.5. Combined Exposure to BPA and BPAF Upregulated Prostate Cell Viability

**Figure 4:**
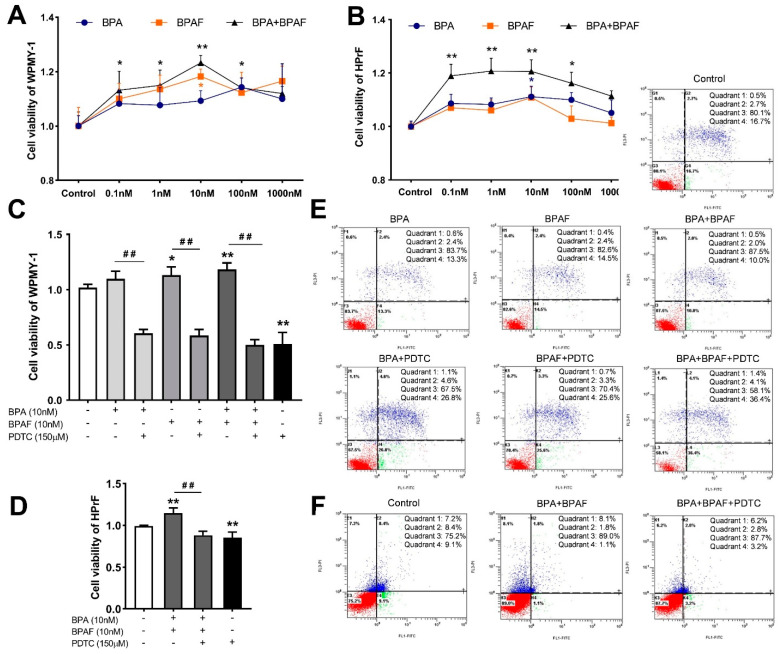
Exposure to BPA, BPAF and PDTC destabilized the viability and apoptosis of prostate cells. Effect of exposure to gradient BPA and BPAF on the viability of WPMY−1 (**A**) and HPrF (**B**). Effect of BPA and BPAF combined with the inhibitor of NF-κB (PDTC) on the viability of WPMY−1 (**C**) and HPrF (**D**). Apoptosis of WPMY−1 (**E**) and HPrF (**F**) that were treated with BPA, BPAF and PDTC. Results are performed as means ± SD, analyzed using ANOVA followed by LSD post hoc test, *n* = 3. Comparison of the individual administration group and the control: * *p* < 0.05, ** *p* < 0.01; comparison of the combined administration group and the individual administration group: ## *p* < 0.01. Quadrant 1: mechanically damaged cells; quadrant 2: FITC+/PI+, late apoptosis of cells; quadrant 3: FITC−/PI−, normal cells; quadrant 4: FITC+/PI−, early apoptosis of cells. WPMY−1: human normal prostate stromal immortalized cell line; HPrF: human prostate fibroblasts.

To identify the superimposed effect of BPA and BPAF, the gradient BPA (0.001–10,000 nM) and BPAF (0.001–10,000 nM) were used to individually and simultaneously treat the human prostate stromal cells and fibroblasts. BPA and BPAF at 0.1–1000 nM were selected as the optimum concentration range for promoting cell viability (Figure 4A,B). The cell survival curves of the human normal prostate stromal immortalized cell line (WPMY−1) and human prostate fibroblasts (HPrF) were increased earlier and decreased later with proliferation, which either peaked or reached plateaus with 10 nM. Of particular note, the facilitated effect of co-exposure to BPA and BPAF on cell proliferation is slightly stronger than that of single exposure.

### 2.6. Inhibition of NF-κB Inhibited Proliferation and Stimulative Apoptosis of Prostate Cells

Based on Counting Kit−8 (CCK−8) results, after the co-treatment with BPA, BPAF and PDTC, the viabilities of WPMY−1 cells were dramatically reduced (Figure 4C). Individual treatment with BPA or BPAF had a slightly weaker proliferation effect on HPrF cells, compared to the co-administration of BPA and BPAF. Therefore, the simultaneous exposure to BPA and BPAF was only selected as the optimal way to detect the effect of NF-κB inhibitor on HPrF viability (Figure 4D). The results of Annexin V-FITC/PI staining manifested that the apoptosic rates of individually BPA- or BPAF-exposed WPMY−1 cells were moderately reduced (Figure 4E). In addition, the apoptosis resistance of the co-treatment of BPA and BPAF was more obvious (Figure 4E,F). Furthermore, inhibition of NF-κB counteracted the disrupted effect of BPA and BPAF and sped up the apoptosis of the prostate cells.

### 2.7. In Vitro Validation of the Modulation of NF-κB Signaling Pathway

**Figure 5:**
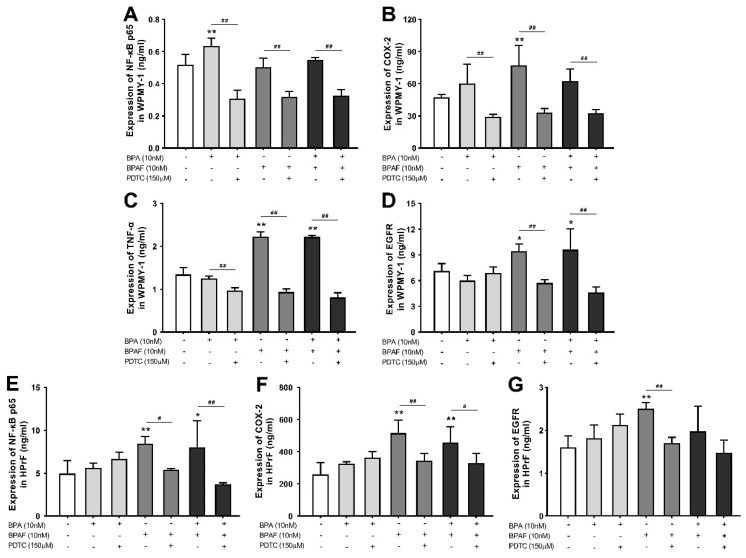
PDTC influenced BPA- and BPAF-facilitated NF-κB p65, COX-2, TNF-α and EGFR expression in prostate cells. Effect of exposure to BPA, BPAF and the inhibitor of NF-κB (PDTC) on the expression of NF-κB p65 (**A**), COX-2 (**B**), TNF-α (**C**) and EGFR (**D**) in WPMY−1 cells and the levels of NF-κB p65 (**E**), COX-2 (**F**) and EGFR (**G**) in HPrF cells. Results are performed as means ± SD, analyzed using ANOVA followed by LSD post hoc test, *n* = 3. Comparison of the individual administration group and the control: * *p* < 0.05, ** *p* < 0.01; comparison of the combined administration group and the individual administration group: # *p* < 0.05, ## *p* < 0.01.

Quantitative detection showed that BPA induced the most significant elevation of NF-κB p65 activity in WPMY−1 cells (Figure 5A). The mixed exposure to BPA and PDTC decreased the expression of NF-κB p65, COX-2 and TNF-α (Figure 5A–C), indicating that BPA may intervene in cell proliferation and apoptosis by activating the NF-κB signaling pathway, thereby stimulating the expression of COX-2 and TNF-α, while EGFR did not show a direct interaction (Figure 5D). In parallel, the expressions of COX-2, TNF-α and EGFR in WPMY−1 cells could be upregulated by individual BPAF treatment or BPA combined with BPAF. In HPrF cells, the exposure to BPAF alone or the co-exposure to BPA and BPAF upmodulated the expressions of NF-κB p65, COX-2 or EGFR, while PDTC led to a decrease in the abnormal expression (Figure 5E–G). Additionally, the content of TNF-α in HPrF cells was not significantly affected by BPA or BPAF.

## 3. Discussion

A growing body of credible evidence has demonstrated that persistent exposure to bisphenol compounds may initiate the abnormal proliferation of germ cells, disrupt the development and function of the endocrine system and even drive malignant transformation [29,30,31,32]. This research was established to investigate the associations between BPAF and prostatic hyperplasia and explore the regulation of the NF-κB signaling pathway in BPA- and BPAF-induced prostatic toxicity. Bisphenols have been reported to induce prostatic urethra, bladder and prostate enlargement in male mice, as well as increase nonvoiding contractions [33], similar to the evaluation of the BPA- and BPAF-stimulated prostatic hyperplasia in this paper. Compared with the control, the ascended weight and organ coefficient of the VP and the histomorphological analysis of the thickened epithelium in the rats of the BPA (90 μg/kg) and BPAF (90 μg/kg) groups intuitively implied the effect of BPA and BPAF for hyperproliferative prostates. Meanwhile, emerging as a proliferation-promoting indicator [34], the ascended rate of positive expression of PCNA solidified the abovementioned speculation about bisphenols’ induction initiating the proliferative status in rat prostates. Combined administration of the inhibitor of NF-κB can reduce the abnormally upmodulated indicators, suggesting that the suppressive NF-κB activity might alleviate the prostatic toxic effects of BPA and BPAF.

Theoretically and mechanistically, the presence of trifluoromethyl in the chemical structure of BPAF could result in a greater ability to bind to estrogen receptors, while we found that the effects of BPA and BPAF on the phenotype of prostatic hyperplasia in rats and the proliferation of human prostate cells were generally similar. It is conceivable that this could be ascribed to the multiplicity of the pathogenesis of the prostate disorders elicited by bisphenols. More specifically, hormone receptors and other signal transduction pathways could jointly mediate the BPA- and BPAF-induced trajectories of prostate development in rats. This may also be interpreted as the metabolism of BPAF to BPAF-G eliminating the agonistic estrogen and antagonistic androgen and thyroid activities of BPAF [35]. Previous findings held that BPA and BPAF have practically identical effects in driving testicular feminization [36]. The estrogenic activity of bisphenol S (BPS) is weaker than that of BPA, while BPS performed a moderately stronger effect on embryonic stem cell differentiation than BPA [37]. These studies indicated that the estrogenic activity of bisphenol compounds is not exactly proportional to their toxic effects. However, n in vitro study showed that the activation of the NF-κB signaling pathway was slightly more sensitive to individual exposure to BPAF than to BPA, which is potentially associated with the heterogeneity of in vivo and in vitro model validation, mirroring the difference in the molecular structures of BPA and BPAF. Overall, we considered that directly equating the estrogen-interfering effects of bisphenol compounds with their reproductive toxicity-inducing effects might be one-sided.

BPH tissue is characterized by an elevated proliferative potential, decreased apoptotic levels and the excessive growth of prostate stromal cells, followed by excessive ductal budding and branching [38,39,40]. In vitro studies have found that individual exposure to BPA or BPAF at 0.1–1000 nM promoted the proliferation of human prostate stromal cells and fibroblasts, which is consistent with the in vivo exploration. The dose response of BPA showed that 0.01–100 nM BPA contributed to the centrosome amplification and anchorage-independent growth of the prostate epithelial cell line RWPE−1 [41]. In addition, low-dose BPA (1 nM) could promote the number of branched structures of the developmental prostate, which is attributed to the BPA-targeted embryonic stem cells that interfere with the directional differentiation into the prostate [42]. The above findings underscored the BPA- or BPAF-induced elevation of prostate cell viability.

The superposition effect of concurrent exposure to multiple environmental estrogens has attracted significant scientific and public attention. Exploring the combined effects of environmental estrogens is instructive for environmental modeling and for a more accurate assessment of the risk of BPA-induced pathological processes in the prostate. Evidence has been reported that the co-exposure of BPA, BPS and BPAF had additive effects on cardiomyocyte differentiation and embryonic stem cell proliferation [37]. The joint treatment of BPA and nonyl phenol (NP) on RWPE−1 cell viability showed significant synergism, which is greater than the effects of single exposure to BPA or NP [43]. This study examined the survival and apoptosis levels of WPMY−1 cells and HPrF cells after exposure to BPA and BPAF alone and in combination, revealing that the conjoint exposure to BPA and BPAF promoted cell proliferation and inhibited cell apoptosis more strongly than exposure to either alone. This could depend principally on the interaction of the above chemicals, amplifying the mutual effect [44].

The inflammatory effects of bisphenols were inextricably linked to the dysregulated tissue hyperplasia, cell proliferation and apoptosis [45,46,47]. The NF-κB signaling pathway, which seemed to be the imperative convergence of proinflammatory factors, has gradually become a research hotspot of BPA-triggered toxicity [48,49]. In the human prostatic epithelium, the inflammasome component NLRP3 activation controlled by NF-κB and ROS modulation could provoke the release of a series of proinflammatory factors and initiate an inflammatory cascade [50]. The BPA- and BPAF-driven ascended expression of and COX-2 in rat VP, as well as in human prostate cells, demonstrated that both of them hold promise as the potential interlocutors in mediating the prostatic hyperplasia disrupted by BPA and BPAF. The expressions of NF-κB p65 and COX-2 were attenuated by the NF-κB inhibition, suggesting a mutual regulatory association between them. Abnormally activated NF-κB and proinflammatory enzyme COX-2 are common in various prostate lesions. Sequence analysis of the 50-flanking region of the *COX-2* gene shows two NF-κB sites [25], denoting that COX-2 expression was affected by NF-κB activity. The NF-κB/COX-2/PGE2 activity negatively regulated by the involvement of the cystic fibrosis transmembrane conductance regulator (CFTR) in human BPH samples provides a mechanistic explanation for the interaction [51].

TNF-α is a common proinflammatory cytokine that can bind with tumor necrosis factor receptor 1 (TNFR1) to incur IκB phosphorylation and the subsequent nuclear translocation of NF-κB through various signaling molecules, so as to modulate inflammatory, anti-apoptotic, and immune responses [52]. This is also the reason for the sensitivity difference between normal human prostate epithelial cells and prostate cancer cells to TNF-α-induced biological responses [53]. EGFR is a transmembrane protein that can be activated by epidermal growth factor (EGF), transforming growth factor-α (TGF-α) and integrin. Regarding the downstream signals of EGFR, EGFR could cause NF-κB signaling pathway hyperactivity by triggering a Ras/MAPK cascade reaction [26] and the intervening PI3K/Akt/mTOR signal phosphorylation [54]. The EGFR-associated NF-κB signaling pathway mediates cell survival, accompanying the abnormal increase in COX-2, TNF-α and Cyclin D1. From our experimental verification in male rats, TNF-α was involved in the regulation of the NF-κB signaling pathway. The abnormally upregulated EGFR expression was associated with BPA and BPAF exposure in hypertrophic VP, and EGFR reduction was regulated by the inhibitor of NF-κB. As a mitogen in keratinocytes and fibroblasts, EGFR activation could induce epithelial–mesenchymal transition (EMT) and NF-κB signaling in prostate epithelial cells, thereby initiating epithelial survival programs and protecting cells from apoptosis [55]. Additionally, the in vitro findings emphasized that the elevated expressions of TNF-α and EGFR influenced by NF-κB activity might be involved in the excessive proliferation of WPMY−1 cells treated with co-exposure to BPA and BPAF. In addition, little is known about the regulatory relationship between TNF-α and NF-κB in BPA- and BPAF-induced prostatic toxicity. Thus, the effect of the inhibition of NF-κB activity on TNF-α expression has the potential to be different between the in vivo and in vitro models. On the basis of the ELISA results in HPrF cells, co-treatment of BPA and BPAF caused the overexpression of EGFR, and PDTC relieved EGFR dysfunction, which consolidated the regulatory relationship between NF-κB and EGFR. Of note, TNF-α might have no significant driver function in the BPA- and BPAF-induced proliferative HPrF cells.

## 4. Materials and Methods

### 4.1. Animal Treatment

Ninety male Sprague-Dawley rats weighing 370 to 400 g and aged 77 to 83 days were purchased from Zhejiang Vital River Laboratory Animal Technology Co., Ltd. (Zhejiang, China). Under a 12 h:12 h light/dark cycle, all animals, with free feeding (Shanghai Shilin Science & Tech Co., Ltd., Shanghai, China) and drinking, were housed in a room maintained at 20–26 °C and 40–70% humidity. All rats were handled following the Guidelines for the Care and Use of Laboratory Animals.

After a 5-day quarantine and acclimatization period, the animals were randomly assigned to nine groups (*n* = 10) according to body weights. The vehicle, BPA (10, 90 µg/kg), BPAF (10, 90 µg/kg) and PDTC (100 mg/kg) were given to the rats by intragastric administration for 4 weeks. The specific groups are named the control, BPA (10 μg/kg), BPA (10 μg/kg) + PDTC, BPA (90 μg/kg), BPA (90 μg/kg) + PDTC, BPAF (10 μg/kg), BPAF (10 μg/kg) + PDTC, BPAF (90 μg/kg) and BPAF (90 μg/kg) + PDTC. After administration, the rats were sacrificed by asphyxiation with carbon dioxide. The ventral lobe of prostate was dissected and weighed. One part was immediately taken for fixation in 10% formalin, while the other part was frozen in liquid nitrogen at −80 °C for protein detection. The weight gain of the rat prostate was evaluated by weighing the VP and calculating 1000 times the ratio of VP weight to the terminal body weight, as the organ coefficient of VP.

### 4.2. Pathological Evaluation

The 48 h-formalin-fixed tissues from VP were dehydrated, cleared, waxed and embedded in paraffin, and then every tissue was cut into 4 μm thick sections using a microtome (Leica, China). The histological sections were immersed in xylene for deparaffinization, transferred to 100% ethanol and follow-up 75% ethanol for rehydration and subsequently stained with hematoxylin and eosin (H&E). The sections were observed and photographed under an inverted microscope (Olympus, Tokyo, Japan). The epithelial height of each sample was measured using image analysis software (Olympus, Tokyo, Japan).

### 4.3. IHC Analysis

The location and semiquantitative analysis of proliferating cell nuclear antigen (PCNA), NF-κB p65, COX-2, TNF-α and EGFR in the prostate sections were evaluated using IHC. After dewaxing and rehydration, the sections were immersed in 0.01 M citrate buffer (pH 6.0), using microwave oven heating for 20 min for antigen retrieval. Endogenous peroxidases and non-specific bindings were, respectively, inhibited with oxidase blocking solution and normal nonimmune serum. Subsequently, the histological sections were incubated with primary antibodies against PCNA (1:100, AF1363, Beyotime, Shanghai, China), p-NF-κB p65 (1:75, AF5881, Beyotime, Shanghai, China), COX-2 (1:75, 31296I11P56, Boster, Wuhan, China), TNF-α (1:250, AF8208, Beyotime, Shanghai, China) and EGFR (1:120, AF5153, Beyotime, Shanghai, China) overnight at 4 °C, separately. The sections were covered with secondary antibodies at 37 °C for 10 min. After the substrate catalysis with streptomyces antibiotic peroxidase solution and staining with 3,3′-diaminobenzidine (DAB) and hematoxylin, the sections were dehydrated, washed and sealed with neutral gum. Eight images were collected from VP of each group, separately. The amount of PCNA and p-NF-κB p65 that predominantly expressed in the nucleus, were respectively used to calculate the positive expression rate that presented as the ratio of the positively expressed nucleus to the total nucleus. In parallel, the semi-quantification levels of COX-2, TNF-α and EGFR that were mainly located in the cytoplasm were evaluated using the IHC profiler plugin of ImageJ software.

### 4.4. Tissue-Based ELISA

The frozen tissues were cut into pieces, weighed, ground in phosphate-buffered saline (PBS) (w:v = 1:10) and centrifuged for homogenate preparation. Based on the instructions of the rat ELISA Kits of NF-κB p65 (ab176648, Abcam, Cambridge, UK), COX-2 (CSB-EL13399r, CUSABIO, Wuhan, China), TNF-α (EK0526, Boster, Wuhan, China) and EGFR (ELR-RGFR-1, RayBiotech, Norcross, GA, USA), the content of these proteins in the prostate could be determined using a microplate reader (Tecan, Männedorf, Switzerland) and i-control software.

### 4.5. Cell Cultivation

Human normal prostate stromal immortalized cell line (WPMY−1) was supplied from Shanghai Zhong Qiao Xin Zhou Biotechnology Co., Ltd. (Shanghai, China), and human prostate fibroblasts (HPrF) were from American Sciencell Company. In an incubator with the atmosphere of 5% CO^2^ and 95% air, the WPMY−1 cells were cultured with DMEM containing 5% fetal bovine serum (FBS) and 1% penicillin-streptomycin (P/S). HPrF cells were cultured in fibroblast medium supplemented with 2% FBS, 1% fibroblast growth supplement (FGS) and 1% P/S.

### 4.6. Cell Proliferation Assay

The WPMY−1 cells and HPrF cells were seeded in 96-well plates, cultured for 24 h and exposed to gradient BPA (0.001–10,000 nM) and BPAF (0.001–10,000 nM) for 48 h (4 × 10^3^ cells/well). CCK−8 assay was used to assess the viability of the prostate cells, to determine the optimum proliferative doses of BPA and BPAF to be given separately and together. Then, the prostate cells were reinoculated and treated with the optimal doses of BPA (10 nM), BPAF (10 nM) and PDTC (150 μM) for 48 h (4 × 10^3^ cells/well); CCK−8 solution (10 μL/well) was added and detected using an enzymatic reader (Thermo Fisher Scientific, Shanghai, China) at 450 nm.

### 4.7. Cell Apoptosis Detection

Both types of prostate cells were inoculated in 6-well plates at the density of 3 × 105 cells/well, cultured for 24 h and treated with BPA (10 nM), BPAF (10 nM) and PDTC (150 μM) for 72 h. After trypsin enzymic digestion and washing with PBS, the cells were resuspended in binding buffer and stained using annexin V-fluorescein isothiocyanate (FITC) and propidium iodide (PI). Thereafter, the apoptosis rate of the cells was analyzed using flow cytometry (Beckman, Fullerton, CA, USA) and CXP analysis software.

### 4.8. Cell-Based ELISA

The procedures of cell inoculation and treatment were the same as the section of cell apoptosis detection. Subsequently, WPMY−1 cells and HPrF cells were collected and lysed with RIPA lysate buffer, following the centrifugation to collect the supernatants as the samples. In accordance with the protocol of the manufacturer of the human ELISA Kits (NF-κB p65, 022214CYF129780224, Jianglaibio, Shanghai, China; COX-2, 022214CYF194700224, Jianglaibio, Shanghai, China; TNF-α, EK0525, Boster, Wuhan, China; EGFR, EK0327, Boster, Wuhan, China), the cell samples were incubated with the corresponding antibodies and colored. Ultimately, the protein levels of the potential targets in the WPMY−1 cells and HPrF cells could be quantified using an enzymatic reader at 450 nm (Thermo Fisher Scientific, Shanghai, China).

### 4.9. Statistical Analysis

The data were expressed in the form of means ± SD, statistically analyzed using IBM SPSS Statistics 26.0 software (SPSS Inc., Chicago, IL, USA) and visually performed using Graphpad Prism 9 software (GraphPad Software, San Diego, CA, USA). After meeting the criteria of normality test and homogeneity of variance test, ANOVA was applied for statistical comparisons, and LSD method was used to analyze the post hoc multiple comparisons. *p* < 0.05 was considered as the statistically significant level.

## 5. Conclusions

In conclusion, our findings comprehensively elucidated that, similar to BPA, sustained exposure to BPAF could evoke prostatic hyperplasia in rats and elevate the viability of human prostate cells. The NF-κB signaling pathway that dominated with NF-κB p65, COX-2, TNF-α and EGFR was judged to participate in the BPA- and BPAF-induced prostatic toxicity. Concomitant treatment with BPA and BPAF had a superimposed effect of stimulating cell proliferation, indicating a potentially deleterious effect of co-exposure to multiple environmental estrogens. In addition, it is unlikely to be appropriate to apply BPAF ubiquitously to consumer products as an alternative to BPA.

## Data Availability

Data available upon request.
